# *Beauveria bassiana* Strains for Biological Control of *Cosmopolites sordidus* (Germ.) (Coleoptera: Curculionidae) in Plantain

**DOI:** 10.1155/2013/184756

**Published:** 2013-09-26

**Authors:** Marilene Fancelli, Alex Batista Dias, Italo Delalibera Júnior, Sandra Cerqueira de Jesus, Antonio Souza do Nascimento, Sebastião de Oliveira e Silva, Ranulfo Correa Caldas, Carlos Alberto da Silva Ledo

**Affiliations:** ^1^Embrapa Cassava & Fruits, C.P. 7, 44380-000 Cruz das Almas, BA, Brazil; ^2^Federal Institute of Education, Science and Technology of Bahia–Campus Catu, R. Barão de Camaçari 118, 48100-000 Catu, BA, Brazil; ^3^ESALQ/USP, C.P. 9, 13418-900 Piracicaba, SP, Brazil; ^4^Embrapa Cassava & Fruits, UFRB, Rua Rui Barbosa 710, 44380-000 Cruz das Almas, BA, Brazil

## Abstract

The objective of this study was to select strains of *Beauveria bassiana* for controlling *Cosmopolites sordidus* (Germ.) in plantain farms (cv. Terra) of the “Recôncavo” and southern regions in the state of Bahia, Brazil. The virulence of 32 *B. bassiana* isolates against *C. sordidus* was determined under laboratory conditions. Three isolates (CNPMF 407, CNPMF 218, and CNPMF 416) were selected for evaluation under field conditions in plantations located in the counties of Mutuípe and Wenceslau Guimarães. Population of *C. sordidus* was estimated every 15 days by using pseudostem traps. The efficiency of the three strains of *B. bassiana* was compared to chemical control (carbofuran, 4g/trap) and absence of control. Carbofuran caused around 90% of adult mortality after 12 months, with a reduction in the population of *C. sordidus* since the first evaluation. A low number of trapped insects was observed in the fungus-treated plots, suggesting the efficiency of the isolates in controlling the *C. sordidus* population. The strain CNPMF 218 was the most efficient in controlling *C. sordidus* adults in both locations, causing around 20% mortality, leading to 40% population size reduction after 12 months.

## 1. Introduction

*Cosmopolites sordidus* (Germ.) is a severe constraint to production in most areas where bananas (including plantains) are cultivated [1]. Although all banana varieties are attacked by the pest under growing conditions, varieties from subgroup “Terra” are the most susceptible ones [2], requiring a great effort to control it.

There is an increasing demand for organically produced foods, contributing to the adoption of ecologically oriented pest control methods. Consequently, reduced pesticide use has become a strong option to protect the environment and human health. Thus, the use of biological control agents, such as entomopathogenic fungi, has been considered a useful alternative to pest control. In fact, *Beauveria bassiana* is one of the most studied entomopathogenic fungi, worldwide, aiming at *C. sordidus* control [1, 3–5].

Several works have been carried out in Brazil under laboratory conditions, aiming at selecting virulent isolates of *B. bassiana* [6–11]. However, biological control of banana borer weevil in Brazil under field conditions has not been consistent, depending on the isolate and on the field-delivery methods [7, 9, 12].

Considering that there is a high genetic variability in *B. bassiana* and possible implications regarding virulence and adaptability to local conditions [11], this paper aimed to select the most suitable isolate(s) under laboratory and field conditions for controlling *C. sordidus* in plantain crops cv. “Terra” located in the “Recôncavo” and southern region of the state of Bahia.

## 2. Material and Methods

This work was carried out under laboratory conditions and in plantain farms (cv. Terra) of the “Recôncavo” and southern region of the state of Bahia, in the counties of Mutuípe (13°14′S, 39°30′W, and 320 m high) (“Recôncavo”) and Wenceslau Guimarães (13°41′S, 39°28′W, and 170 m high) (southern region), presenting average annual temperatures of 23.4°C and 24°C, respectively. There are no data about local rainfall and humidity.

The insects used in laboratory experiments were collected from pseudostem traps in the plantain crop cv. Terra. Groups formed by 50 specimens were maintained in quarantine at 25 ± 1°C, 80 ± 10% RH, and 14 : 10 h, L : D, during 15 days, on Petri dishes (15 cm diameter). The adults were fed pseudostem slices of plantain cv. Terra.

*Selection of Isolates of Beauveria bassiana under Laboratory Conditions.* The isolates of *B. bassiana* (Table 1) were inoculated onto adults of *C. sordidus* and reisolated in PDA medium (Potato Dextrose Agar) to be used as inoculum for the production of fungus inside “Roux” bottles containing 100 g of autoclaved rice (40 minutes at 120°C). Fifteen days later, 5 mL from a suspension at 10^8^ conidia/mL was sprayed on 50 adults of *C. sordidus* on Petri dishes (15 cm diameter), by using a DeVilbiss atomiser. Five minutes after the spraying, the insects were placed into plastic boxes (9 × 12 × 11 cm), in the proportion of 10 insects per box. The boxes were maintained at 25 ± 1°C, 95 ± 5% RH, and 14 : 10 h, L : D. The experimental design used was completely randomized with five replicates. The variables observed were daily mortality and TL_50_. Dead insects were individualised in humid chambers for confirmation of mortality. These data were submitted to analysis of variance and the means were compared by the Tukey test (*P* ≤ 0.05). The isolates that caused mortality higher than 50% were grouped by the Tukey test as well (*P* ≤ 0.05), according to TL_50_ values. The data were transformed to $\sqrt{x+0.5}$. Analysis was performed by using SAS Software (1989).

*Population Fluctuation of C. sordidus.* This study was undertaken to assess the insect population before control in two farms: one located in Mutuípe (from December to February) and the other one located in Wenceslau Guimarães (from March to February). Two 5 ha areas in each county cultivated with plantain “Terra” were split into 1 ha subplots. The criteria used for choosing the areas were plant growth uniformity, age, cultural practices, and pest management (in this case, noninterference in pest population). The adult population of *C. sordidus* was assessed by pseudostem traps consisting of 40 cm pseudostem pieces, split lengthwise in two halves. Twenty traps/ha were randomly distributed every 15 days, and placed near the mat. The insect assessments were carried out 15 days after the traps were distributed, by counting and releasing the insects assessed and by destroying the old traps. 

*Inoculum Preparation for Field Conditions.* Transparent polypropylene bags (35 cm × 22 cm) containing 100 g of autoclaved rice (30 minutes at 120°C) were inoculated with 5 mL of a suspension at 10^8^ conidia/mL of *B. bassiana* [13]. The bags were shaken for a better inoculum distribution and maintained in the growth chamber under environmental conditions from 12 to 15 days. 

*Evaluation of Isolates under Field Conditions.* The treatments evaluated consisted of three isolates of *B. bassiana* previously selected from laboratory studies. The evaluation was performed in the same areas used for population fluctuation studies. The suspensions, prepared one day before application, were maintained in a refrigerator. The suspension was obtained by grinding 300 g of rice plus *B. bassiana* for three minutes in a domestic liquefier, adding 1.000 mL of distilled water and four drops of surfactant. As the suspension showed a viscous consistency, a brush was used to apply it on the surface of the pseudostem traps. For the purpose of controlling the pest, 40 traps were used per ha, randomly distributed in a central area of the plot (20 m × 20 m). The traps were replaced fortnightly, when a new application was performed and the number of insects was recorded (alive and dead adults of *C. sordidus*). Control treatment (i.e., absence of pesticide application) consisted of using only pseudostem traps without any control practice, following the same procedure cited above. Chemical control plots were treated with 4 g/trap carbofuran, following the same procedures mentioned for the previous treatments. The alive and dead insect average data were transformed to $\sqrt{x}$ and submitted to analysis of variance and the Tukey test (*P* ≤ 0.05) for comparison between averages.

## 3. Results and Discussion

**Figure 1:**
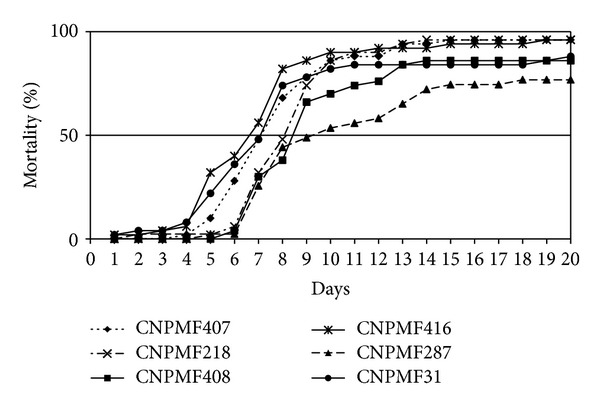
Cumulative mortality for adults of *C. sordidus* caused by four isolates of *Beauveria bassiana* under laboratory conditions (25 ± 1°C, 95 ± 5% RH, and 14 h photophase). Cruz das Almas, Bahia, Brazil.

*Selection of Isolates of Beauveria bassiana under Laboratory Conditions.* Isolates of *B. bassiana* caused from 14% to 96% mortality in *C. sordidus* adults (Table 1), despite pulverization of adults not being considered the best method for *C. sordidus* [14]. For the control treatment, 4% of mortality was recorded. The recorded mortality rates for 13 isolates were higher than 64%. Regarding TL_50_, isolate CNPMF 416 showed the lowest value (6.6 days), differing from isolates CNPMF 326 (12.8 days) and IBCB-66 (14.7 days) (Table 1). Isolates CNPMF 416, CNPMF 407, CNPMF 31, CNPMF 218, and CNPMF 408 also caused high mortality rates and TL_50_ values did not differ from the lowest value observed (Table 1 and Figure 1). Based on these results, the isolates selected for field studies were CNPMF 416, CNPMF 407, and CNPMF 218. These isolates had not been isolated from adults of *C. sordidus*. As shown in Table 1, they were originally isolated from species of Curculionidae collected in the “Recôncavo” region of the state of Bahia. Recordings from studies conducted on this subject [7, 8] showed that the virulence of isolates of *B. bassiana* against *C. sordidus* is not directly associated with the inoculum source. 

**Figure 2:**
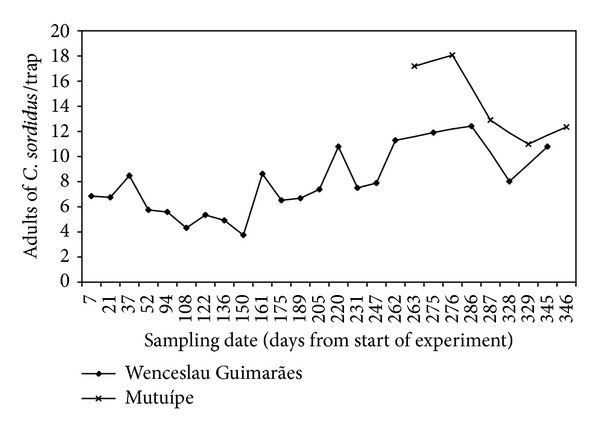
Number of live adults of *C. sordidus* trapped in plantain cv. Terra in Mutuípe and Wenceslau Guimarães, Bahia, Brazil.

*Population Fluctuation of C. sordidus.* The population average for adults of *C. sordidus* in Mutuípe was 14.3 insects/trap (Figure 2) (ranging between 11 and 18 insects/trap). For *Metamasius hemipterus* L., the maximum value collected was up to 1.0 insect/trap. In Wenceslau Guimarães, the average was 7.6 adults of *C. sordidus*/trap (ranging between 4 and 12 insects/trap) (Figure 2). For *M. hemipterus*, the values observed were from 1.4 to 2.1 insects/trap. *M. hemipterus* is not considered a main banana pest [15], but some records show that it enhances *B. bassiana* in banana crops [7, 16]. However, in the present case, this might have not occurred as the number of insects collected was low (averages of 0.4 and 2.0 insects/trap in Mutuípe and Wenceslau Guimarães, resp.). Biotic and abiotic factors have been reported affecting the population dynamics of *C. sordidus* [10]. 

**Figure 3:**
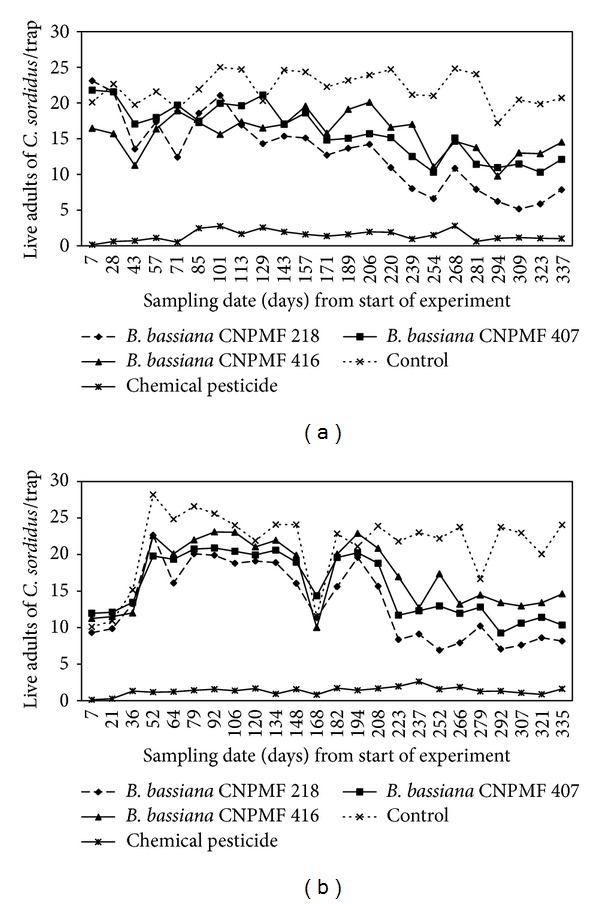
Number of live adults of *C. sordidus* trapped in plantain cv. Terra under different pest management practices. (a) Mutuípe, Bahia, Brazil. (b) Wenceslau Guimarães, Bahia, Brazil.

**Figure 4:**
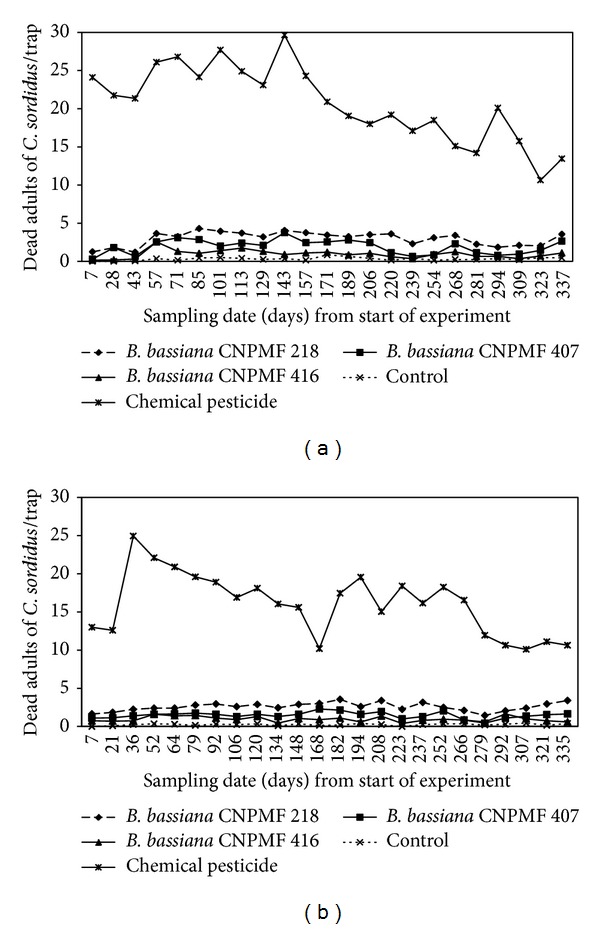
Number of dead adults of *C. sordidus* trapped in plantain cv. Terra under different pest management practices. (a) Mutuípe, Bahia, Brazil. (b) Wenceslau Guimarães, Bahia, Brazil.

*Evaluation of Isolates under Field Conditions.* Population levels of live adults of *C. sordidus* in Mutuípe and Wenceslau Guimarães showed smaller ranges in areas under chemical control (2.6 and 2.5 adults, resp.) than in *B. bassiana*-treated areas (10.3 to 17.9 and 11.6 to 15.7, resp.) (Figure 3). A reduction in adult capture for all treatments, except for chemical control, was observed in Wenceslau Guimaraes around 168 days of sampling (Figure 3). The initial average for live adults was lower in Wenceslau Guimarães (10.6) than in Mutuípe (20.4). The biological treatments and chemical control reduced the population of *C. sordidus* in both areas. Regarding dead adults, the highest values were registered for chemical control (Figure 4). However, mainly in Mutuípe, a higher amplitude between the values (19 adults/trap) was observed, ranging from 2.4 to 3.4 in the treatments with fungus, with the control mean being 0.8. In Wenceslau Guimarães, the amplitude varied from 1.2 to 2.1 for the isolates of *B. bassiana*, being equal to 14.8 and 0.8, chemical control and control, respectively. There was a tendency to reduction in the number of dead adults collected in the chemical treatment, over time.

**Figure 5:**
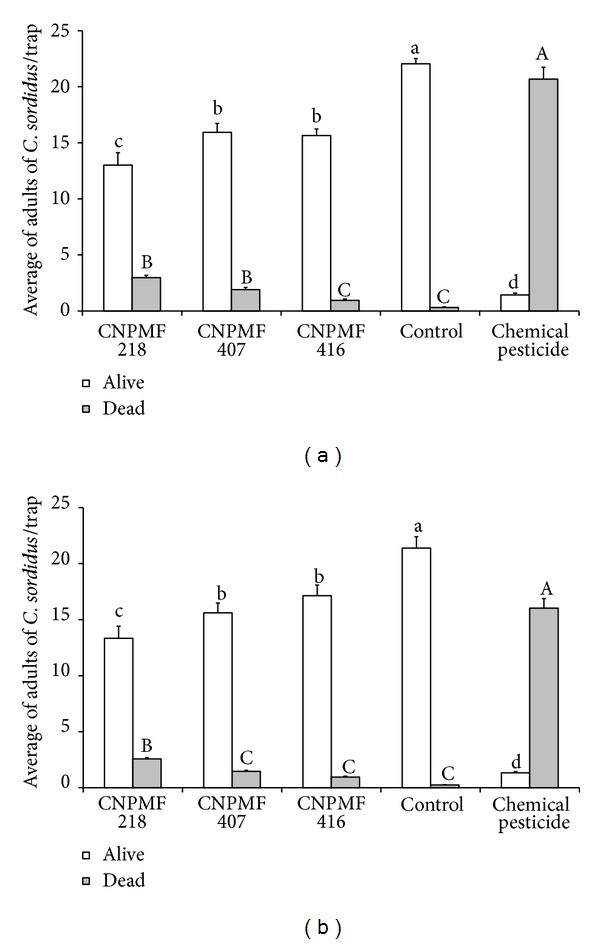
Average of live and dead adults of *C. sordidus* trapped in plantain cv. Terra under different pest management practices. (a) Mutuípe, Bahia, Brazil. (b) Wenceslau Guimarães, Bahia, Brazil. Values followed by the same lower-case letters for live adults and upper-case letters for dead adults do not differ by the Tukey test (*P* ≤ 0.05).

In both areas treated with the entomopathogenic fungus, the number of live adults was lower than in the control (Figure 5). In Mutuípe, the lowest average (13.0 live adults) (*P* ≤ 0.05) was observed for *B. bassiana* CNPMF 218. The values registered for isolates CNPMF 407 and CNPMF 416 did not differ. In Wenceslau Guimarães, the smallest value was observed for isolate CNPMF 218 (13.3 live adults), although it did not differ from CNPMF 407 (15.6 live adults). The averages for live adults at the chemical controlled area (1.4 and 1.3 for Mutuípe and Wenceslau Guimarães, resp.) (*P* ≤ 0.05) were the lowest.

Significant differences were observed among the treatments, regarding the dead adults. The three isolates of *B. bassiana* differed significantly in Mutuípe. Isolate CNPMF 218 showed higher mortality (2.98) (*P* ≤ 0.05) than the others, but all the isolates differed from the control (Figure 5). In Wenceslau Guimarães, the isolates tested differed significantly from the control. The highest value (2.6 dead adults) (*P* ≤ 0.05) was obtained for isolate CNPMF 218. Isolates CNPMF 407 and CNPMF 416 did not differ from each other, showing averages of 1.5 and 1.0 dead adults, respectively (*P* ≤ 0.05). Higher mortality was found for chemical control in both localities (20.7 and 16.0 dead adults/trap in Mutuípe and Wenceslau Guimarães, resp.) (*P* ≤ 0.05).

Considering the numbers of live adults trapped over time, a tendency towards reduced values for the gap between the beginning and end of the evaluations was observed in the treatments under biological control in both places (Figure 3). For the dead adults in the area under chemical control, a tendency towards reduced values was observed from the beginning to the end of the evaluations (Figure 4). The possibility of reinfestation by insects coming from non-treated neighbouring areas must be considered. Since the plots presented similar conditions in relation to cultural management, cultivar, plant age, and insect population, the results indicated that the entomopathogenic fungus reduces the pest population in the plots under biological control. Thus, the lower number of live adults and the consistency in the number of dead adults trapped at each evaluation in the areas submitted to biological control, as compared to the control in both places, may contribute to reduced damage caused by the insects, characterizing the biological control as a feasible lifelong pest control strategy. The mortality registered for the entomopathogenic fungus treatments was lower than that found for the chemical control. However, due to its mode of action, this value may undermine the effect of *B. bassiana*. As the fungal development is slow, some adults contaminated by the fungus would be expected to develop the disease and die, but this was not evaluated. On the other hand, the insects, after contact with conidia, may remain alive and help disperse the entomopathogen, even into nontreated areas. This trait allows the fungus to become an effective mortality agent of *C. sordidus*, as it naturally occurs in Florida [17]. In addition, it is reasonable to consider that weevils should not die in pseudostem traps. Studies developed by Godonou et al. [4] confirmed that banana weevil borer adults artificially infected by *B. bassiana* can move distances up to 18 m, although most of the dead adults, with signs of infection, were located up to 3 m from the release point. Besides that, it is also remarkable that the efficiency of traps for assessing *C. sordidus* population might depend on several factors [1, 18]. However, considering the aim of this work and that the same criteria were applied for all the treatments, this variation might have been minimized over time. Based on that, it can be stated that the method used to apply the fungus was adequate and did not corroborate Mesquita [7], who observed low infection rates (around 5%) of *C. sordidus* by *B. bassiana*, using pseudostem traps immersed in a conidial suspension.

Isolate CNPMF 218 was the most effective for *C. sordidus* control in both locations (Mutuípe and Wenceslau Guimarães) (Figure 5 and Table 2), although the others also reduced the number of trapped adults, compared with the control. The value observed for *C. sordidus* mortality (20.2%), caused by isolate CNPMF 218 (Table 2), was similar to that registered by Khan & Gangapersad [19], in Trinidad and Tobago (20%). Several authors mentioned reductions in the insect population caused by *B. bassiana* [4, 5, 9, 12], although comparisons are difficult because different methodologies have been used. Efforts to develop a standardized methodology should be made, by monitoring *B. bassiana* application under integrated management of *C. sordidus*. Batista Filho et al. [9] observed a population reduction of 61% for adult insects by applying *B. bassiana* (50 mL/pseudostem trap) at 1 × 10^9^ conidia/mL. This work presented reductions ranging from 29.0 to 41.3% and from 18.3 to 35.6%, for Mutuípe and Wenceslau Guimarães, respectively. It must be emphasized that this work used a different concentration from that used by Batista Filho et al. [9] and at a different climatic condition. Although those values are lower than those reported for chemical control, the biological control effect must not be considered to be local.

As mentioned before, studies conducted on biological control under field conditions in Brazil have reported a great variation in results due to several factors (strain, variety, climate, and methodology of fungus application among them). Particularly for the local conditions tested in this paper, it should be mentioned that most of the banana farms are small family based production units in a low input agriculture. Furthermore, they exhibit a strong dependence on chemicals to control *C. sordidus* and consequently to improve banana yield. Concerning the control of *C. sordidus*, the selection of an effective strain adapted to local conditions will contribute to develop a cooperative project on biological control of the pest. Thus, the biological control applied is adequate for sustainable production system and shows several advantages compared to the chemical control, consisting in an ecologically oriented pest control method that can contribute for agricultural sustainability. Besides that, there is still a need for developing a low-cost method of *B. bassiana* delivery under field conditions [11]. *Beauveria bassiana* may be a relevant tool in the integrated management of banana weevil borer in “Recôncavo” and southern Bahia. Isolate CNPMF 218 was the most effective for *C. sordidus* control in plantain cv. Terra, both in Mutuípe and Wenceslau Guimarães. 

## Figures and Tables

**Table 1:** Mortality and TL_50_ (±SE) for adults of *C. sordidus* caused by different isolates of *Beauveria bassiana* (25 ± 1°C, 95 ± 5% RH, and 14 h photophase). Cruz das Almas, Bahia, Brazil.

Strain order number	Strain name	Host	Source	Date	Mortality^1^ (%)	TL_50_ (days)
1	CNPMF 407	*Metamasius hemipterus* L. (Col.: Curculionidae)	Nazaré-BA	19/08/94	96.0 ± 2.7 a	7.4 ± 0.3 ab
12	CNPMF 218	*Metamasius hemipterus* L. (Col.: Curculionidae)	Cruz das Almas-BA	09/04/97	96.0 ± 5.7 a	8.2 ± 0.4 abc
24	CNPMF 416	*Pseudopiazurus papayanus* (Col.: Curculionidae)	Cruz das Almas-BA	02/08/95	96.0 ± 3.1 a	6.6 ± 0.6 a
25	CNPMF 31	Unknown	Unknown	25/03/83	88.0 ± 8.6 ab	7.0 ± 0.5 ab
2	CNPMF 408	*Diploschema rotundicolle* (Col.: Cerambycidae)	Unknown	19/09/94	86.0 ± 9.9 abc	8.4 ± 0.4 abc
5	CNPMF 287	*Cosmopolites sordidus* (Col.: Curculionidae)	Wenceslau Guimarães-BA	12/06/97	79.5 ± 9.9 abcd	10.8 ± 2.0 abc
21	CNPMF 02	Unknown	Espírito Santo	02/05/82	74.0 ± 9.4 abcde	8.4 ± 0.4 abc
4	EPAGRI 01	Unknown	Santa Catarina	23/05/92	73.6 ± 5.4 abcdef	10.4 ± 1.0 abc
15	CNPMF 412	Membracidae on soursop	Nova Soure-BA	05/10/94	72.0 ± 5.7 abcdef	9.8 ± 1.8 abc
19	CNPMF 414	*Cosmopolites sordidus* (Col.: Curculionidae)	Nazaré-BA	10/12/96	70.0 ± 8.7 abcdef	11.0 ± 2.0 abc
14	CNPMF 20	Unknown	Unknown	27/02/86	68.0 ± 9.7 abcdefg	9.2 ± 1.7 abc
22	CNPMF 04	Unknown	Mogi das Cruzes-SP	29/06/95	68.0 ± 2.7 abcdefg	12.4 ± 2.4 abc
8	CNPMF 17	*Cosmopolites sordidus* (Col.: Curculionidae)	Cruz das Almas-BA	08/11/85	64.1 ± 7.2 abcdefg	11.2 ± 0.6 abc
20	643	Unknown	Cuiabá-MT	31/10/96	58.0 ± 8.2 bcdefgh	9.8 ± 0.7 abc
23	CNPMF 415	*Cosmopolites sordidus* (Col.: Curculionidae)	Vicência-PE	07/12/90	56.0 ± 9.6 bcdefgh	9.3 ± 0.8 abc
31	CNPMF 326	*Cosmopolites sordidus* (Col.: Curculionidae)	Nazaré-BA	22/07/97	56.0 ± 2.7 bcdefgh	12.8 ± 1.5 bc
26	CNPMF 01	Unknown	Unknown	17/06/84	54.0 ± 0.0 bcdefgh	9.7 ± 2.0 abc
6	CNPMF 259	Heteroptera on *Malpighia *	Cruz das Almas-BA	16/05/97	52.4 ± 4.5 cdefgh	11.0 ± 3.1 abc
7	CNPMF 410	*Cosmopolites sordidus* (Col.: Curculionidae)	Cruz das Almas-BA	10/10/95	52.1 ± 7.9 cdefgh	10.7 ± 1.5 abc
10	IBCB-66	*Hypothenemus hampei* (Col.: Scolytidae)	São José do Rio Pardo-SP	05/09/94	51.6 ± 8.9 cdefghi	14.7 ± 1.1 c
32	CNPMF 418	*Cosmopolites sordidus* (Col.: Curculionidae)	Cruz das Almas-BA	11/06/93	48.0 ± 7.6 defghij	
13	CNPMF 411	*Cosmopolites sordidus* (Col.: Curculionidae)	Nazaré-BA	10/06/96	46.6 ± 7.4 defghij	
3	CNPMF 409	Membracidae on soursop (Membracidae)	Nova Soure-BA	17/08/92	42.1 ± 12.1 efghij	
17	CNPMF 08	Unknown on citrus	Unknown	13/11/87	40.0 ± 2.7 efghij	
28	CNPMF 417	Unknown	Unknown	30/02/94	38.0 ± 4.2 fghijk	
30	604	Unknown	Cuiabá-MT	31/10/96	38.0 ± 11.5 fghijk	
11	CNPMF 06	Unknown	Cruz das Almas-BA	10/05/95	33.1 ± 7.6 ghijk	
29	CNPMF 18	Unknown	Unknown	27/02/94	28.0 ± 4.2 hijk	
18	CNPMF 413	Unknown	Unknown	27/02/86	16.0 ± 7.4 ijk	
27	CNPMF 419	Membracidae on papaya	Nova Soure-BA	18/07/92	16.0 ± 5.5 ijk	
9	447	Unknown	Cuiabá-MT	31/10/96	14.0 ± 7.6 jk	
control					4.0 ± 4.2 k	

*F*					14.6	2.9

CV (%)					26.1	11.7

^1^Averages followed by distinct letters differ by the Tukey test (*P* ≤ 0.05).

**Table 2:** Mortality and population reduction for adults of *C. sordidus *(±SE) trapped in plantain cv. Terra under different pest management practices. Mutuípe and Wenceslau Guimarães, Bahia, Brazil.

Treatments	Mortality^1^ (%)		Population reduction^2^ (%)	
	Mutuípe	Wenceslau Guimarães	Mutuípe	Wenceslau Guimarães
*Beauveria bassiana* CNPMF 218	20.2 ± 1.4	17.6 ± 1.2	41.3 ± 4.9	35.6 ± 4.8
*Beauveria bassiana* CNPMF 407	10.5 ± 0.9	8.9 ± 0.5	27.4 ± 3.8	23.8 ± 4.8
*Beauveria bassiana* CNPMF 416	5.6 ± 0.6	5.5 ± 0.4	29.0 ± 2.4	18.3 ± 3.4
Control	1.4 ± 0.2	1.1 ± 0.1	—	—
Chemical pesticide	93.4 ± 0.7	92.2 ± 0.6	93.6 ± 0.7	93.9 ± 0.5

^1^(Total of dead insects/total of live insects + dead insects found in traps in each treatment) × 100 per evaluation.

^2^[1 − (Total live insects trapped in each treatment/total live insects trapped by no control treatment)] × 100 per evaluation.

## References

[1] Gold CS, Pena JE, Karamura EB (2001). Biology and integrated pest management for the banana weevil *Cosmopolites sordidus* (Germar) (Coleoptera: Curculionidae). *Integrated Pest Management Reviews*.

[2] Fancelli M, Alves ÉJ, Alves ÉJ (2001). Principais pragas da cultura. *Cultivo de Bananeira Tipo Terra*.

[3] Delattre P, Jean-Bart A (1978). Activités des champignons entomopathogenes (Fungi Imperfecti) sur les adults de *Cosmopolites sordidus* Germ. (Coleoptera: Curculionidae). *Turrialba*.

[4] Godonou I, Green KR, Oduro KA, Lomer CJ, Afreh-Nuamah K (2000). Field evaluation of selected formulations of *Beauveria bassiana* for the management of the banana weevil (*Cosmopolites sordidus*) on plantain (Musa spp., AAB group). *Biocontrol Science and Technology*.

[5] Nankinga CM, Moore D (2000). Reduction of banana weevil populations using different formulations of the entomopathogenic fungus *Beauveria bassiana*. *Biocontrol Science and Technology*.

[6] Batista Filho A, Camargo LMPCA, Myazaki I, Cruz BPB, Oliveira DA (1987). Controle biológico do moleque da bananeira (*Cosmopolites sordidus*, Germar, 1824) pelo uso de fungos entomógenos, no laboratório. *Biológico*.

[7] Mesquita ALM (1987). Controle biológico das brocas da bananeira *Cosmopolites sordidus* (Germar, 1824) e *Metamasius hemipterus* (Linne, 1764) com fungos entomógenos. *Reunion de la Acorbat*.

[8] Busoli AC, Fernandes OA, Tayra O (1989). Controle da broca da bananeira *Cosmopolites sordidus* Germar 1824 (Coleoptera, Curculionidae) através dos fungos entomopatogênicos *Beauveria bassiana* (Bals.) Vuill. e *Metarhizium anisopliae* (Metsch.) Sorok. (Hyphomycetes). *Anais da Sociedade Entomológica do Brasil*.

[9] Batista Filho A, Sato ME, Leite LG, Raga A, Prada WA (1991). Utilização de *Beauveria bassiana* (Bals.) Vuill. no controle do moleque da bananeira *Cosmopolites sordidus* Germar, 1824 (Coleoptera: Curculionidae). *The Revista Brasileira de Fruticultura*.

[10] Prestes TMV, Zanini A, Alves LFA, Batista Filho A, Rohde C (2006). Aspectos ecológicos da população de *Cosmopolites sordidus*, (Germar) (Coleoptera: Curculionidae) em São Miguel do Iguaçu, PR. *Semina: Ciências Agrárias*.

[11] Lopes RB, Michereff-Filho M, Tigano MS (2011). Virulence and horizontal transmission of selected Brazilian strains of *Beauveria bassiana* against cosmopolites sordidus under laboratory conditions. *Bulletin of Insectology*.

[12] Batista Filho A, Leite LG, Raga A, Sato ME (1995). Enhanced activity of *Beauveria bassiana* (Bals.) Vuill associated with mineral oil against *Cosmopolites sordidus* (Germar) adults. *Anais da Sociedade Entomológica do Brasil*.

[13] Alves SB (1998). *Controle Microbiano De Insetos*.

[14] Lopez EA, Neves PMOJ, Almeida VP, Tamiozo G, Fancelli M (2010). Métodos de inoculação e virulência de *Beauveria bassiana* (Bals.) Vuill. a *Cosmopolites sordidus* (Germar). *Semina: Ciências Agrárias*.

[15] Fancelli M (2012). *Metamasius hemipterus* L. como praga de bananeiras cv. Terra. *Revista Brasileira de Fruticultura*.

[16] Pauli G, Biaggioni Lopes R, Damatto ER, Mascarin GM (2011). Falsa broca aumenta disseminação de *Beauveria bassiana* em populações de campo da broca-do-rizoma da bananeira. *Ciência Rural*.

[17] Pena JE, Gilbin-Davis RM, Duncan R (1995). Impact of indigenous *Beauveria bassiana* (Balsamo) Vuillemin on banana weevil and rotten sugarcane weevil (Coleoptera: Curculionidae) populations in banana in Florida. *Journal of Agricultural Entomology*.

[18] Gold CS, Okech SH, Nokoe S (2002). Evaluation of pseudostem trapping as a control measure against banana weevil, *Cosmopolites sordidus* (Coleoptera: Curculionidae) in Uganda. *Bulletin of Entomological Research*.

[19] Khan A, Gangapersad G (2001). Comparison of the effectiveness of three entomopathogenic fungi in the management of the banana borer weevil, *Cosmopolites sordidus* (Germar) (Coleoptera: Curculionidae). *International Pest Control*.

